# Role of Inflammasomes in HIV-1 and Drug Abuse Mediated Neuroinflammaging

**DOI:** 10.3390/cells9081857

**Published:** 2020-08-08

**Authors:** Susmita Sil, Fang Niu, Ernest T. Chivero, Seema Singh, Palsamy Periyasamy, Shilpa Buch

**Affiliations:** Department of Pharmacology and Experimental Neuroscience, University of Nebraska Medical Center, Omaha, NE 68198, USA; susmita.sil@unmc.edu (S.S.); fang.niu@unmc.edu (F.N.); ernest.chivero@unmc.edu (E.T.C.); seema.singh@unmc.edu (S.S.)

**Keywords:** HIV-1, drug abuse, inflammasomes, neuroinflammation, aging, proinflammatory cytokines

## Abstract

Despite the effectiveness of combined antiretroviral therapy (cART) in suppressing virus replication, chronic inflammation remains one of the cardinal features intersecting HIV-1, cART, drug abuse, and likely contributes to the accelerated neurocognitive decline and aging in people living with HIV-1 (PLWH) that abuse drugs. It is also estimated that ~30–60% of PLWH on cART develop cognitive deficits associated with HIV-1-associated neurocognitive disorders (HAND), with symptomatology ranging from asymptomatic to mild, neurocognitive impairments. Adding further complexity to HAND is the comorbidity of drug abuse in PLWH involving activated immune responses and the release of neurotoxins, which, in turn, mediate neuroinflammation. Premature or accelerated aging is another feature of drug abusing PLWH on cART regimes. Emerging studies implicate the role of HIV-1/HIV-1 proteins, cART, and abused drugs in altering the inflammasome signaling in the central nervous system (CNS) cells. It is thus likely that exposure of these cells to HIV-1/HIV-1 proteins, cART, and/or abused drugs could have synergistic/additive effects on the activation of inflammasomes, in turn, leading to exacerbated neuroinflammation, ultimately resulting in premature aging referred to as “inflammaging” In this review, we summarize the current knowledge of inflammasome activation, neuroinflammation, and aging in central nervous system (CNS) cells such as microglia, astrocytes, and neurons in the context of HIV-1 and drug abuse.

## 1. Introduction

The advent of combined antiretroviral therapies (cART) for HIV-1 infection in the mid-1990s has been one of modern medicines most dramatic triumphant stories. Essentially, once a death sentence—with a lifespan of ~18 months post-diagnosis—HIV-1 infection, managed with cART, now has transformed into a mostly manageable, chronic condition like diabetes. A person starting cART today can expect to live reasonably for another 30 to 50 years, and often well into older age. Paradoxically, however, with success has come a new set of challenges facing both the patients and health professionals. Despite the successful control of viremia, as the infected individuals start early initiation of cART and as a result, continue to live longer, they are at risk of developing age-related comorbidities such as cognitive impairments, cardiovascular disease, certain cancers, kidney and liver disease and osteoporosis, decades ahead of their HIV-1-negative counterparts. While some with well-treated HIV-1 can expect a healthy life expectancy, others cannot, especially in the older population, where excess mortality increases with age, and survival time is significantly lower than in the uninfected population [1,2]. Elegant clinical studies have demonstrated that chronic HIV-1 infection results in a five-year increase in biological age [3]. The health needs of the increasing number of HIV-1-positive Americans who are reaching older ages have become a significant public health issue. According to the Centers for Disease Control and Prevention, the number of those over age 50 living with HIV-1 is rapidly escalating. For example, while in 2001 there were ~17% of people diagnosed with HIV-1 infection that were age 50 or over, this number almost doubled (~33%) in 2009 and is expected to be >70% in 2020 [4]. These trends are mirrored globally. Adding further complexity to the problem in the aging population is the onset of accompanying physiological changes that have been implicated in increasing the risk for several comorbidities in this group of individuals, and this is further exacerbated in PLWH on cART regimens [5]. Whether this is because of a chronic effect of the residual HIV-1/HIV-1 proteins, cART treatment itself, increased dependence on pain medications, or by physiological alterations already set in place by HIV-1 infection remains unknown. Given the persistent, chronic low-level smoldering inflammation present in PLWH on cART regimens [5,6,7], it is speculated that this ongoing immune activation likely contributes to aging [8,9,10,11,12,13,14]. In addition to the effects on the immune system and inflammation, HIV-1 also infects as well as affects the brain. Prior to the development of effective treatment, ~8–15% of PLWH developed a severe cognitive disorder-HIV-1-associated dementia, the incidence of which in the post-cART era has now declined to ~2% [14,15,16,17,18]. Intriguingly, despite effective cART treatment and undetectable plasma viral levels, ongoing low level of chronic immune activation and inflammation culminates into accompanying comorbidities commonly associated with the aging population, including vascular atherosclerosis, metabolic syndrome, and neurodegeneration. As a result, there continue to be largely increased numbers of PLWH that are afflicted with HIV-1-associated neurocognitive disorders (HAND) or NeuroHIV [19,20,21,22,23,24,25,26]. According to epidemiological data, it is estimated that ~30–60% of PLWH on cART develop cognitive deficits associated with HAND, with symptomatology ranging from asymptomatic to mild, motor neurocognitive impairments. HAND is highly prevalent and remains a grave complication, despite successful cART, severely impacting the life quality of PLWH [27,28,29,30]. A recent elegant review by Lanman et al. (2019) has described the neurotoxic effects of antiretrovirals and its potential role in inflammation and premature aging in PLWH [31].

Adding additional complexity to HAND is the comorbidity of substance abuse (opiates, cocaine, and methamphetamine) in PLWH [32,33,34,35]. In fact, drug use and HIV-1 have been inextricably linked as intertwined epidemics in the 1980s. Today, illicit drug use is a crucial driver of HIV-1 infection across the globe [36,37]. Neuroimaging research conducted prior to active treatment or on untreated individuals has suggested that HIV-1 accelerates aging of the brain and that comorbidity of drug abuse exacerbates neurological aging among PLWH [38,39,40]. It is reported that of those afflicted with HAND, at least 30% will have a history of drug abuse. Drug use and addiction can hasten the progression of HIV-1 and its consequences, especially in the brain. Clinical research indicates that drug use and addiction could increase the viral load, accelerate disease progression, and worsen AIDS-related mortality even among patients on cART regimens [32]. In addition, people with substance use disorders are less likely to adhere to the life-saving HIV-1 medication regimen [41], thereby leading to the activation of virus blips during their illness. Drugs can make it easier for HIV-1 to enter the brain and trigger an immune response and initiate glial activation and release of neurotoxins, which, in turn, mediate neuroinflammation [42]. HIV-1-induced inflammation in the brain, thus underlies the neurocognitive disorders or NeuroHIV and is closely linked to accelerated brain aging, manifesting as a severe complication of HIV-1 infection [43,44,45,46,47,48]. Glial cell activation involving brain astrocytes and microglia as well as infiltrating monocyte/macrophages, resulting in ensuing neuroinflammation, has been recognized as an underlying hallmark feature of HAND [49,50,51]. Intriguingly, smoldering, and persistent low-level inflammation both in the periphery as well as in the central nervous system (CNS) is a key feature driving the severity of CNS complications. NeuroHIV is challenging to diagnose and treat since other factors—such as aging, drug abuse, drug addiction, and psychiatric illnesses—are common and can produce similar cognitive symptoms [26]. Premature or accelerated aging is another feature observed in drug abusing PLWH who are on cART regimens. Emerging studies implicate the role of HIV-1/HIV-1 proteins, cART, and abused drugs in altering the inflammasome signaling in cells of the CNS such as microglia, astrocytes, and neurons. It is thus likely that exposure of these cells to HIV-1/HIV-1 proteins, cART and/or abused drugs can have synergistic/additive effects in activation of inflammasomes, thus contributing to exacerbated neuroinflammation, ultimately culminating into “inflammaging”. In this review, we summarize the current knowledge of inflammasome activation, neuroinflammation, and aging in CNS cells such as microglia, astrocytes, and neurons.

## 2. Inflammaging

**Figure 1:**
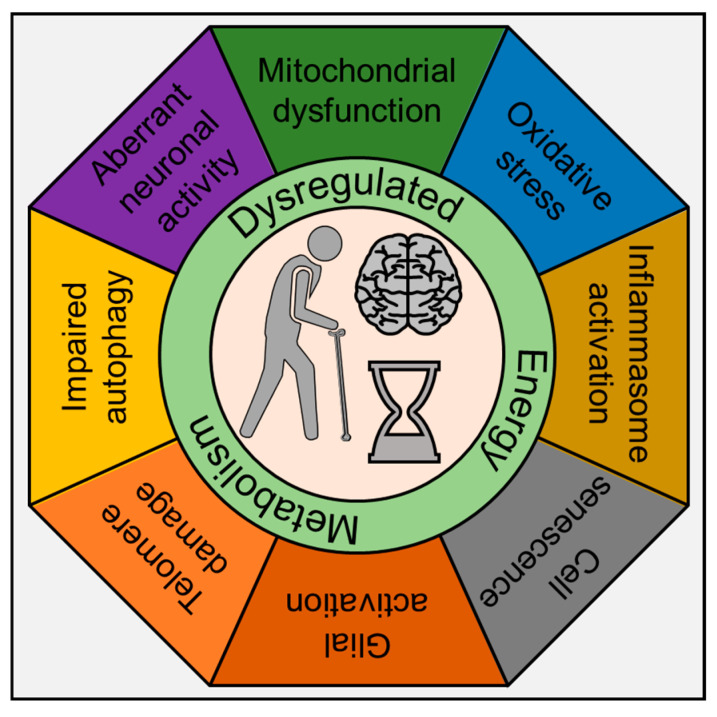
Schematic diagram depicts the various cellular pathways implicated in inflammaging.

Recent years have seen an explosive surge in research on inflammation and its effects on aging, now referred to as “inflammaging” [52]. As stated above, life-long infection with HIV-1, coupled with early initiation and dependence on cART and comorbidity of drug abuse, results in a persistent, slowly fueling inflammatory milieu in the brain that accumulates over time and results in premature or accelerated aging and neurodegeneration. Recent studies have assessed the expression of inflammation and aging-related markers in the PBMCs of PLWH treated with cART regimen and have reported increased expression of IL6, IL18, and CXCL10 with a concomitant decrease in aging-associated markers such as p53, PAI-1 and IGFBP-3 [53]. In the HIV-1-infected, cART treated, and drug-abusing population, these neurodegenerative, cognitive impairments are seen decades earlier than the normal healthy counterparts (uninfected and drug naïve individuals). While there is extensive literature on inflammaging in response to lifestyle stressors during normal aging, there is a knowledge gap in our understanding of how HIV-1, cART, and drug abuse contribute to accelerated aging. Furthermore, considering that by the end of 2020, >70% of infected people will be 50 years or older—there is an urgent need in the field to link how inflammation mediated by these agents either individually or in combination (HIV-1, cART, and drug abuse) contributes to the aging phenotype in the whole organism. Studies aimed at assessing the mechanism(s) underlying normal aging have interrogated the brain at both the cellular and molecular levels and found many of the same hallmarks of aging as evident in other tissues [3,53,54,55,56,57,58]. These hallmarks include inflammation; mitochondrial dysfunction; intracellular accumulation of oxidatively damaged proteins, nucleic acids, and lipids; dysregulated energy metabolism; impaired cellular “waste disposal” mechanisms (autophagy–lysosome and proteasome functionality); impaired adaptive stress response signaling; compromised DNA repair; dysregulated neuronal Ca^2+^ handling; telomere damage; and cell senescence (Figure 1).

## 3. Inflammasomes

Inflammasomes are cytosolic multiprotein complexes that function as molecular platforms for the activation of the inflammatory caspases, including caspase-1 and -11 [59,60,61,62]. The caspases are usually present in an inactive form and undergo conformational changes and activation upon their recruitment into the multiprotein complex [63,64]. Activated caspase-1 or -11 are known cysteine proteases that can specifically cleave their substrates after aspartate residues [65]. IL1β and IL18 are among the known substrates of these inflammatory caspases that are also recruited to the multiprotein complex and are cleaved, ultimately leading to functional modulation of signaling pathways involved in various cellular processes such as programmed cell death, differentiation, and cell proliferation [66].

The nucleotide-binding domain, leucine-rich repeat-containing proteins (NLRs) that are critical for inflammasome formation, are composed of three separate domains. The *C*-terminal region is made up of variable numbers of leucine-rich repeats (LRRs) that are thought to autoinhibit NLR in the resting state and undergo stimuli-induced conformational changes [67]. The central nucleotide-binding and oligomerization (NACHT) domain has been shown to oligomerize following inflammasome activation in the presence of nucleotides such as ATP and is essential for NLR function [68,69,70]. The *N*-terminal domain consisting of either a Pyrin or caspase activation and recruitment domain (CARD) are essential for the recruitment of caspases involving protein-protein interactions [67,71]. Nearly 20 NLR inflammasomes have been identified in humans with the most investigated inflammasomes being NLRP1, NLRP3, NLRP6, NLRC4, and NLRP12 [72]. Inflammasomes are broadly classified into two categories—the proinflammatory inflammasomes (e.g., NLRP3 and NLRC4) and the anti-inflammatory inflammasomes (e.g., NLRP12, NLRX1, NLRC3, and NLRC5). Taking the case of NLRP3 as an example, inflammasome formation involves the recruitment of ASC through binding of the Pyrin domain followed by recruitment of pro-caspase-1 through CARD–CARD homotypic interactions [67]. For inflammasomes that lack a Pyrin domain, such as NLRC4, recent evidence has demonstrated that it can directly recruit caspase-1 through a *C*- or *N*-terminal CARD domain [68]. Following recruitment to the inflammasome complex, pro-caspase-1 oligomerizes and is cleaved by autocatalysis, resulting in the release of the active catalytic p20 and p10 caspase-1 fragments and subsequent processing of precursor IL1β into its biologically active 17 kDa fragment [68].

For the activation of the inflammasome machinery, the host first recognizes either pathogen-associated molecular patterns (PAMPs) expressed by microbial pathogens or danger-associated molecular patterns (DAMPs) that are produced by host cells [59,72,73,74,75,76]. To date, several molecules have been described that can serve as PAMPS or DAMPS, which, among others, also include HIV-1 viral proteins and drugs of abuse. These latter agents will be discussed in this review in more detail. Cells within the CNS such as microglia, macrophages, and astrocytes have been shown to express NLRs and are involved in inflammasome activation and ensuing neuroinflammation. Among these cells, microglia and astrocytes have been shown to express NLRP3 [77,78] and NLRC4 [78,79]. Additionally, astrocytes have also been shown to express NLRP2 [80], and neurons express NLRP1 [81,82,83]. We will briefly describe each of these inflammasomes and their activation in the context of HIV-1 and drug abuse studies.

### 3.1. NLRP1 Inflammasome

NLRP1 is expressed in several types of immune cells as well as in non-hematopoietic tissues in the peripheral system [83] and in the neurons in the CNS [81,82,83]. The human NLRP1 has a unique structural layout consisting of an *N*-terminal CARD, a central NACHT, LRR, FIIND, and a *C*-terminal CARD [60]. NLRP1 has been shown to interact with caspase-1 and -5, resulting in the formation of multiprotein complexes in cell-free lysates that is necessary for IL1β/IL18 processing and pyroptosis [62]. Other studies have demonstrated that NLRP1 can interact with caspase-2 and -9 to facilitate cell death via the apoptosome [84], suggesting its involvement in multiple cell death pathways. NLRP1 is activated by the anthrax lethal toxin resulting in the release of proinflammatory cytokines, which facilitates the clearance of the bacterial infection of *Bacillus anthracis* [85,86]. It has also been shown that HIV-1 replication is associated with increased NLRP1 inflammasome activation, IL1β, and IL18 expression in the gut-associated lymphoid tissue and peripheral blood of HIV-1 infected persons [87]. In the context of substance abuse, studies in mice administrated 5% ethanol for 5 weeks, demonstrated increased expression of NLRP1, along with the increased expression of NLRP3, ASC, and proinflammatory cytokines, thus underscoring the ability of alcohol to activate the inflammasomes [88]. Recent studies from the same group have shown that inhibition of the inflammasome signaling cascade using VX765 and MCC950 reduced alcohol consumption in female mice, further validating the involvement of alcohol in activation of the inflammasome pathway and ensuing neuroinflammation [89]. In another study in an ex vivo model of organotypic hippocampal-entorhinal cortex brain slice cultures, the inflammasome signaling was shown to mediate ethanol mediated inhibition of hippocampal neurogenesis [90]. These authors demonstrated that ethanol-mediated impairment of neurogenesis involved increased expression of NALP1, NALP3, and IL1β in both neurons and astrocytes. Furthermore, blockade of IL1β synthesis with the inflammasome inhibitors such as parthenolide and Bay11708 reversed ethanol mediated inhibition of neurogenesis [90]. In the same study, the authors also demonstrated upregulated expression of NLRP1 and NLRP3 in the hippocampus of human postmortem brain tissues of alcohol-using subjects, thereby supporting a role for alcohol in inflammasome signaling [90].

### 3.2. NLRP3 Inflammasome

NLRP3 is primarily expressed by myeloid cells. In the CNS, microglia and infiltrating macrophages have been shown to express NLRP3 [77]. Structurally, NLRP3 consists of an *N*-terminal pyrin domain, a central NBD, and a *C*-terminal LRR [60,91]. The apparent lack of a CARD in NLRP3 implies that NLRP3 cannot recruit procaspase-1 except in the presence of the adaptor molecule ASC [60]. It has been demonstrated that NLRP3 interacts with ASC via pyrin domain homophilic interactions [92]. Several endogenous and exogenous agonists such as pathogen-associated molecule patterns (PAMPs), environmental agents (asbestos, silica, UV radiation), and self-derived sterile activators (ATP, cholesterol, amyloid β) are known to activate the NLRP3 inflammasome [60]. In the context of HIV-1, Walsh et al. have demonstrated that HIV-1 infection induced the expression of caspase 1, NLRP3, IL1β, and IL18 in the cerebral white matter of PLWH [93,94]. Other studies in human monocytes have shown that HIV-1 infection-induced production of IL1β via the NLRP3 inflammasome-dependent mechanism(s) [95]. In addition, HIV-1 Tat protein has also been reported to activate the mouse microglial NLRP3 inflammasome [96]. To further support the involvement of NLRP3-inflammasome in HIV-1 pathogenesis, in a study involving 150 HIV-1-infected Brazilian subjects and 158 healthy controls, Pontillo et al., (2012) demonstrated that single nucleotide polymorphisms within NLRP3 (rs10754558) and IL1β (rs1143634) genes were significantly associated with HIV-1 infection [97]. Together these studies provide evidence for the role of the NLRP3 inflammasome during HIV-1 infection. In the context of drug abuse, cocaine has been shown to upregulate the expression levels of NLRP3 in macrophages infected with HIV-1 [98]. The production of reactive oxygen species (ROS) was also increased in cocaine-exposed macrophages, thus suggesting an upstream role for ROS in cocaine-mediated priming of the NLRP3 inflammasome. Similar to NLRP1, levels of NLRP3 have also been reported to be upregulated in animal models of alcohol administration [88] as well as in postmortem human samples [90].

### 3.3. NLRC4 Inflammasome

NLRC4 is expressed in cells of hematopoietic origin and consists of an *N*-terminal CARD, a central NBD domain, and *C*-terminal LRRs [60]. Due to the presence of a CARD domain in NLRC4, it interacts directly with procaspase-1 via homotypic CARD interactions, leading to the cleavage of caspase-1. Activation of the NLRC4 inflammasome leads to IL1β and IL18 secretion and pyroptotic cell death. The NLRC4 inflammasome is specifically activated by intracellular flagellin [99,100]. The specificity of flagellin in the activation of NLRC4 was shown when a mutant strain of Legionella that do not express flagellin failed to activate caspase-1 [101]. In the context of HIV-1, one study demonstrated that activation of the NLRC4 pathway by flagellin rescued the NLRP3 inflammasome defect in dendritic cells from HIV-1-infected patients suggesting that NLRC4 activation could be utilized as a possible future adjuvant in immunocompromised individuals who exhibit poor response to immunization [102].

### 3.4. NLRC5 Inflammasome

NLRC5 is abundantly expressed in the mucosal epithelial cells and in the brain [103] and is emerging as a key regulator of immune responses to combat intruding microbes [104]. Recent studies have shown that NLRC5 negatively regulates NF-κB signaling via direct binding to the NF-κB regulators IKKα/IKKβ, thereby preventing both the recruitment of IKKγ as well as nuclear translocation of NF-κB [105,106]. NF-κB is known to acts as a key point of convergence for several signaling pathways that are pertinent for the antiviral response and subsequent activation of the adaptive immune responses [107]. Consequently, downregulation of NLRC5 could represent a unique mechanism by which HIV-1 Tat interferes with the host defense system, leading, in turn, to persistent inflammation and cellular activation. It is also reported that exposure of mouse microglial cells to recombinant HIV-1 Tat protein demonstrated downregulation of NLRC5 protein with simultaneous activation of NF-κB signaling, thereby suggesting a possible association of NLRC5-mediated activation of NF-κB signaling axis in microglia [108]. However, the expression profile of NLRC5 on cART, as well as drugs of abuse, remains unclear.

### 3.5. AIM2 Inflammasome

AIM2 (Absent in melanoma 2) is a cytoplasmic sensor of double-stranded DNA which localizes to the cytosol. AIM2 consists of an *N*-terminal pyrin domain and a *C*-terminal HIN200 domain and binds dsDNA via the HIN200 domain [60]. The pyrin domain of AIM2 is hypothesized to interact with the pyrin domain of ASC, leading to caspase-1 recruitment to the complex to form a stable and competent inflammasome [60]. AIM2 does not discriminate between the origins of DNA it detects but relies on its presence in the cytosol to initiate a response. Activation of the AIM2 inflammasome is mediated by ds DNA binding where the DNA and protein heteroduplex recruits ASC and caspase 1. Similar to NLR inflammasomes, the AIM2 inflammasome results in IL1β and IL18 secretion and cell death. AIM2 plays a role in the responses to viruses, including HIV-1. In one study, HIV-1 infected macrophages exposed to cocaine exhibited significant upregulation of AIM2 mRNA and IL1β [98].

## 4. Neuroinflammation in the Context of HIV-1 and Drug Abuse

Inflammation within the brain and spinal cord, termed as neuroinflammation, is driven by activation of immune cells in the CNS, leading to the production of cytokines, chemokines, reactive oxygen species, and secondary messengers [109]. Several studies have implicated proinflammatory cytokines (IL1β, IL6, and TNFα), chemokines (CCL2, CCL5, CXCL1), secondary messengers (NO and prostaglandins) and reactive oxygen species (ROS) as common inducers of neuroinflammation [45,93,96,109]. The outcome of a neuroinflammatory response, however, is a double-edged sword with both positive effects such as tissue regeneration or protection against infection and also adverse effects such as cell death, cellular senescence, and chronic neurodegeneration, depending of course on the context, duration, and course of the primary stimuli. In this review, we will focus on neuroinflammation in the context of HIV-1 and drug abuse.

Increased neuroinflammation has been reported in PLWH involving upregulation of microglial activation detected in studies using positron emission tomography or magnetic resonance imaging involving assessment of the 18 kDa translocator protein (TSPO), a marker of microglial activation [110,111,112,113,114]. White matter damage and neuronal injury have also been reported in subjects with HAND [115]. In another study that analyzed autopsy tissues from PLWH and varying degrees of neurocognitive impairment but without HIV-1 encephalitis (HIVE), neuroinflammation was determined by the status of activated microglia determined by CD163, CD16, and HLA-DR expression, with many microglia exhibiting a rounded or ramified morphology with thickened processes, classically associated with activation [116].

Substance abuse involving psychostimulants such as methamphetamine and cocaine has also been shown to be associated with neuroinflammation. For example, methamphetamine exposure has been shown to increase microglial activation and reactive oxygen species production in mice, and this involved the sigma-1 receptor, MAPK, and Akt pathways [117]. In another study, microglia from SIV-infected macaques treated with methamphetamine were shown to exhibit increased expression of proinflammatory genes encoding chemokines, chemokine receptors, and NOD-Like receptor pathways genes [118]. Exposure of murine microglia to cocaine has also been shown to increase the expression of TNFα and IL6 genes while also inducing oxidative stress and activation of the TLR2 signaling pathway [119,120]. These studies demonstrate the role of drugs of abuse in the induction of neuroinflammation, which has thus the potential to contribute to sustained, systemic, low-grade inflammation—a critical factor in driving the aging process.

## 5. Role of Microglia—Inflammasomes and Aging

Microglia, the immunocompetent and phagocytic cells, reside both in the white and gray matter and comprise approximately 10% of the central nervous system (CNS) cells. Microglia express many pattern-recognition receptors (PRRs) including nucleotide-binding and oligomerization domain (NOD)-like receptors, Toll-like receptors (TLR) that detect the pathogen-associated molecular patterns (PAMPs) or tissue damage-associated molecular patterns (DAMPs), ultimately leading to the production of the proinflammatory cytokine, IL1β, which can induce neuroinflammation [121]. Alterations in microglial functionality are implicated during brain development and the aging process [122]. Furthermore, as the primary source of proinflammatory cytokines, microglia are important mediators of neuroinflammation and can modulate a broad spectrum of cellular responses. Although microglial numbers and morphology (increased cell body and decreased length of the process) are altered in the brains of PLWH [123], the role of microglia in mediating premature aging in PLWH in the context of drug abuse remains unclear. There is an increased incidence of mild cognitive impairment in PLWH in the era of cART. Neuroinflammation is a signature of HAND in PLWH [112]. In HAND patients and drug abusers, microglia exhibit an activated phenotype with increased production of inflammatory cytokines [111,124,125]. Increased expression of proinflammatory cytokines has been shown to play a key role in cognition, memory and learning, and sensory functions of the inflammasome components [74,126]. Upregulation of various inflammasomes including NLRP1, NLRP3, NLRC4, NLRC5 has been reported in age-related progressive neurodegenerative diseases, autoimmune disorders, and in brain injury [74,127,128,129].

According to Furman et al., “inflammaging” is related to inflammasome activation [130]. Inflammasomes play a significant role in neuroinflammation owing to their ability to regulate the activation of various inflammatory responses. Notably, inflammasomes have been shown to be activated during aging and in age-related CNS diseases, accelerating the process of senility and CNS disorders [131]. Several studies have confirmed the key role of microglia in aging people and in aging-related diseases [132,133,134,135]. Recent emerging evidence indicates that HIV-1/HIV-1 proteins and drugs of abuse such as psychostimulants, morphine, and alcohol each or in combinations activate inflammasomes in microglia, thereby contributing to neuroinflammation [123,125]. It can thus be speculated that activated inflammasome mediated upregulation of neuroinflammation in microglia plays a significant role in the premature aging process during chronic HIV-1 infection and its comorbidity with drugs of abuse. Several reports suggested that the accumulation of various endogenous metabolic DAMPs signals related to endoplasmic reticulum (ER) stress [136], mitochondrial dysfunction [137] lysosomal damage [138] lipid deposits [139] or K^+^ efflux [140] are associated with increased inflammasome activation and likely in the activation of aged microglia. There is also evidence of increased mitochondrial microRNAs (let-7b, miR-146a, miR-133b, miR-106a, miR-19b, miR-20a, miR-34a, miR-181a, and miR-221) and cellular microRNA (miR-146a, miR-34a, and miR-181a) in inflammasome-dependent inflammaging [96,108,141,142,143]. Mechanism(s) underlying HIV-1/HIV-1 proteins and/or drug abuse in mediating aging of microglia likely involves dysregulated TLR-signaling [144], activation of the NLRP3 signaling and downregulation of NLRC5 inflammasome [96,108].

**Figure 2:**
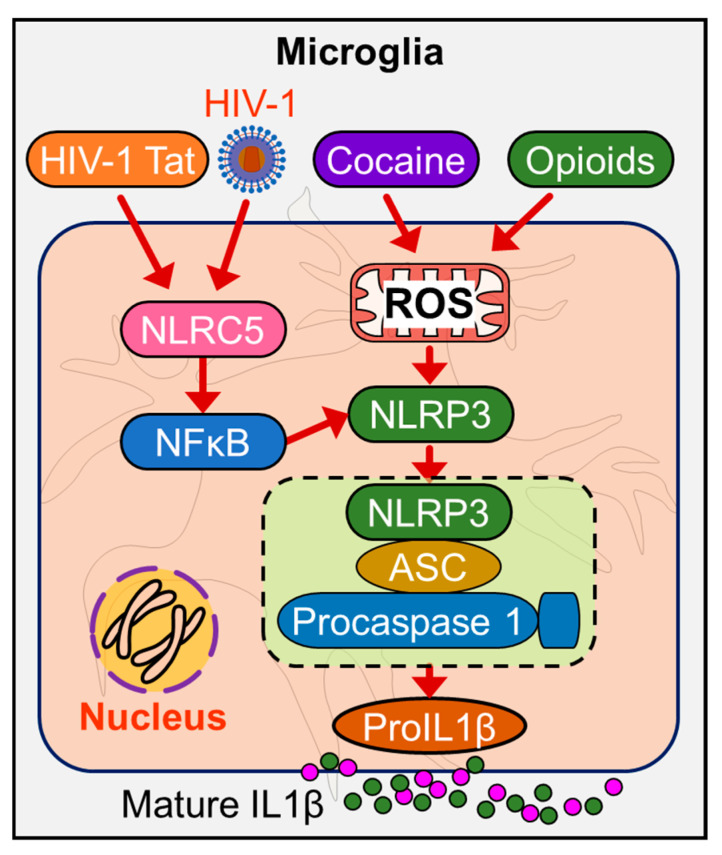
Schematic diagram depicts the mechanism(s) by which HIV-1/HIV-1 Tat and drug abuse modulate the NLRP3 and NLRC5 inflammasome signaling in the microglial cells. HIV-1/HIV-1 proteins, and drugs of abuse co-operatively activate microglia via: a) downregulated expression of NLRC5 resulting in activation of NF-κB (signal 1), leading, in turn, to transcriptional upregulation of NLRP3, and IL1β, and b) induction of ROS-mediated mitochondrial dysfunction (signal 2), which, in turn, induces assembly of NLRP3 inflammasome, leading to increased cleavage and secretion of mature IL1β, ultimately culminating in neuroinflammation and ensuing inflammaging.

Current evidence indicates that cocaine potentiates activation of ROS in HIV-1-infected macrophages, and this is accompanied by the upregulation of inflammasome genes [98]. Microglia are known to express the potassium channel (Kv). HIV-1 protein gp120 has shown to upregulate the expression of voltage-gated K+ (Kv1.3) channels in microglia [145]. Many in vitro studies, including those from our group, have attempted to unveil the molecular pathways underlying HIV-1/HIV-1 proteins and drugs of abuse mediated microglial activation and have shown the involvement of ER stress, mitochondrial dysfunction, ROS production, and dysregulated miRNAs in the activation of inflammasomes in microglia [146,147,148,149]. Our group demonstrated that HIV-1 Tat-mediated microglial NLRC5 activation involves the miRNA-34a-NLRC5-NF-κB signaling axis [108]. Further, miR-223 has also been shown to play a significant role in NLRP3 inflammasome priming in response to HIV-1 Tat protein leading, in turn, to microglial activation [96] and persistent neuroinflammation (Figure 2).

A recent clinical study has suggested the miRNA signature underlying cognitive deficits and alcohol use disorder in PLWH [150]. A study on feline immunodeficiency virus infection has also demonstrated the activation of multiple inflammasome-associated genes in microglia, and this was shown to be accompanied by neuronal loss in the cerebral cortex as well as neurological deficits [93]. Taken together, HIV-1 in the context of drug abuser can mediate premature aging which is regulated by activation of the microglial inflammasome involving both “signal 1” and “signal 2”, in turn, leading to neuroinflammation and associated neuronal damage and cell death, which manifests as accelerated or premature aging.

## 6. Role of Astrocytes—Inflammasomes and Aging

Astrocytes, one of the most abundant glial cells in the brain, play critical roles in neurogenesis through the formation, maintenance, and pruning of synapses and by potentiating neuronal survival through the uptake and release of glutamate, scavenging of free radicals, and via the production of cytokines and nitric oxide to maintain neuronal homeostasis [151]. In addition, astrocytes also induce innate immune responses via a limited TLR repertoire [152,153]. Contrary to other CNS cells, astrocytes are resilient to death receptor-induced apoptosis but are susceptible to various inflammatory insults [154,155]. Activated astrocytes, by regulating various signaling pathways, can exert potent proinflammatory culminating into neuroinflammation [156,157]. As described above, the ongoing neuroinflammation is reinforced by key innate immune sensor(s) for danger signals referred to as inflammasomes. In addition to microglia, astrocytes also express various members of the inflammasome family, as reported in various disease models [78,80,129,158,159,160,161,162,163,164,165,166,167,168,169,170,171,172,173]. NLRP1 is the first reported inflammasome expressed in the rat primary cortical astrocytes that is activated by purinergic receptors [174]. Recent literature has demonstrated differential expression of various inflammasomes in astrocytes, with most of them related to various neurodegenerative diseases as well as aging [175,176,177,178].

In addition to microglia, astrocytes have also been considered as an HIV-1 cellular reservoir within the CNS [179,180,181,182,183]. However, the outcome of HIV-1 infection on these cells is less clear [181]. Recently, Ojeda and his colleagues reported the activation of NLRP3 inflammasome in human primary astrocytes infected with HIV-1 and suggested new and divergent relationships of astrocytes productively infected with HIV-1 with the neighboring non-productively infected astrocytes [164]. These authors further claimed that HIV-1-infected astrocytes exhibit mitophagy as a critical mechanism to subvert imminent cell death. In contrast, the astrocytes that are affected as a result of the bystander effect demonstrated activation of the NLRP3 inflammasome, which, in turn, was related to mitochondrial damage [164]. In the doxycycline-inducible, astrocyte-specific HIV-1 Tat transgenic mice (iTat) model, it has been reported that long-term expression of HIV-1 Tat in the brain manifested poor memory and motor function outcomes along with brain region- and gender-specific dysregulation of neuropathological changes, similar to age-related changes involving increased astrocyte activation and altered synaptic plasticity—the ability of neurons to bring about changes in the connections between neuronal networks in response to use or disuse [184]. HIV-1 infection has been shown to accelerate the biological aging process of HIV-1-infected individuals by ~five years in blood cells [3] and ~seven years in the brain [56,57]. Overall, it is evident that the presence of HIV-1 Tat protein in PLWH under cART [58] raises the likelihood of accelerated aging in PLWH. In addition to HIV-1 infection and HIV-1 proteins, long term usage of antiretrovirals has also been reported to induce astrocytes senescence in human fetal astrocytes exposed to clinically relevant combinations of antiretrovirals in vitro [185] thus implicating that cART-mediated astrocyte senescence induced injury to the surrounding neuronal tissue. It is also reported that human fetal astrocytes infected with HIV-1 demonstrated astrocyte senescence, and this was further validated in ex vivo astrocytes isolated from HIV-1-infected humanized mice as well as in the brains of HIV-1 transgenic rats [178]. The precise mechanisms underlying astrocyte senescence, however, remains unclear [186].

Drugs of abuse have also been known to activate inflammasome signaling and aging in astrocytes, culminating in neuroinflammaging. Recently, methamphetamine-mediated neuroinflammatory responses were reported in cerebral organoids comprising of astrocytes [187]. In this study, exposure of cerebral organoids to methamphetamine elicited both novel astrocyte-specific gene expression networks as well as activation of the NLRP1 inflammasome. Furthermore, the authors also found an association between the NLRP1 inflammasome activation and methamphetamine-induced astrocytosis [187]. In another study in astrocytes exposure of cells to HIV-1 gp120 and morphine resulted in cleavage of caspase-1 and pro-IL1β, thereby confirming the combinatorial effects of HIV-1 gp120 and morphine on canonical inflammasome activation and astrocytosis [188]. However, more studies are warranted to demonstrate the interactions of inflammasome signaling and astrocyte senescence in the context of HIV-1 and drug abuse and ensuing neuroinflammaging.

## 7. Role of Neurons—Inflammasomes and Aging

Studies have shown that structural and metabolic parameters of the brain decline at relatively younger ages in HIV-1-infected Apoε4 carriers leading to the cognitive deficits and inflammation [189,190]. It is important since Apoε4 allele is linked with increased risk of cognitive impairment [191]. Overall, however, neuropsychological studies on the cohort of PLWH have produced conflicting results regarding the impact of ApoE4 or the Apoε4 allele on the course of cognition. During normal aging, ApoE4 contributes to cognitive impairment, and the same effect is observed in the aging HIV-1 population. In vitro studies have demonstrated alterations in neuronal gene expression following exposure of cells to HIV-1. The ApoE4 phenotype potentiates diminished neurogenesis at the gene expression level [192], by direct transcriptional repression for some genes [193]. Thus, the regulation of genes by ApoE4 related to neurogenesis in the brain of PLWH could likely regulate aging and cognitive reserve. Studies on serum from PLWH have demonstrated an association between the inflammatory markers such as CXCL1 and TGF-α and shortening of telomere length. Furthermore, IL10RA on neurons was found to be associated with decreased telomere length [194], thus indicating an inflammaging.

### Role of Extracellular Vesicles (EVs) in Aging

Aging is characterized by loss of regenerative capacity and is the predominant risk factor for several diseases and conditions affecting the lifespan of an individual. EVs have been shown to play important roles in aging. Recent study has shown that EVs derived from healthy hypothalamic stem/progenitor cells when implanted into the hypothalamus of mid-aged mice resulted in slowing of aging. Further, functional hypothalamic stem/progenitor cells have also been shown to release EVs into the cerebrospinal fluid (CSF), which could contribute to slowing of the aging process [195]. Interestingly, it was also observed that senescent cells release increased numbers of EVs [196,197,198], leading to senescence-associated secretory phenotype. EVs have also been shown to serve as biomarkers of aging. EVs from the serum of aged rats have been reported to carry reduced levels of CD63 and increased acetylcholinesterase, miR-96, miR-182, miR-183 compared to young controls and these levels were altered by exercise [199]. Moreover, miRNA-183-5p mimic transfected in bone marrow cells, showed reduced cell proliferation and increased senescence, thus indicating that bone marrow EVs from aged animals could suppress osteogenesis [199]. Another interesting study showed that there was a decrease in number of plasma EVs in older individuals and that these EVs were readily taken up by B cells, which induced increased expression of MHC-II on monocytes compared to EVs from younger individuals. This study thus showed that circulating EVs in aged individuals had the potential to modulate immune responses [200].

**Figure 3:**
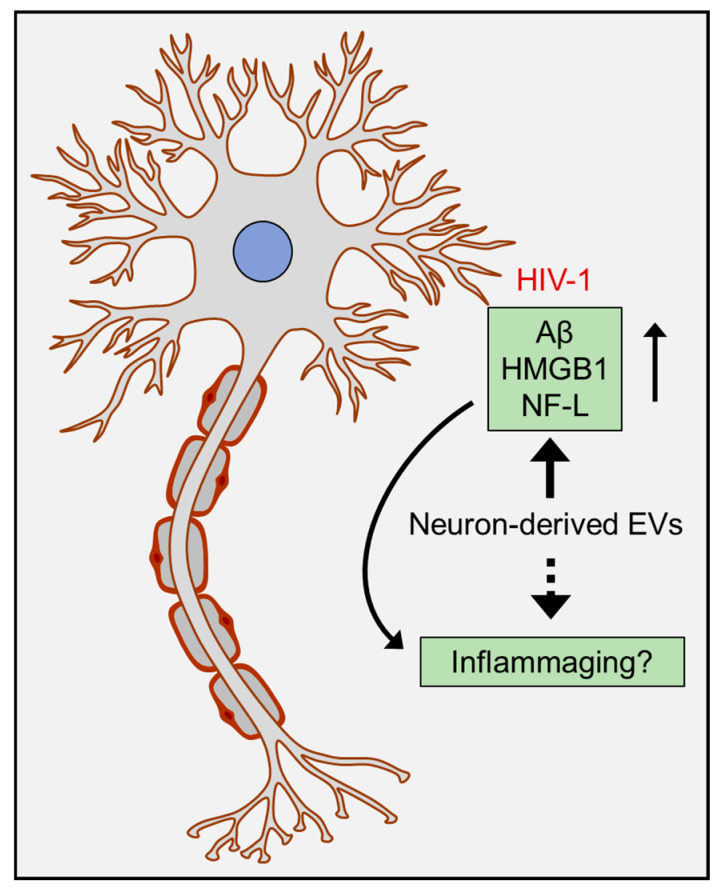
Alzheimer’s like neuropathology in HIV-1 leads to inflammaging: Amyloids that are synthesized by the neurons and non-neuronal cells in the presence of HIV-1 in the CNS can lead to neuroinflammation either directly, or via the amyloid carrying EV cargoes. Accumulation of amyloids causes tissue damage and leads to inflammation and infiltration of immune cells into the brain, in turn, resulting in the activation of glial cells and production of proinflammatory mediators. furthermore, these pathways can trigger a vicious cycle leading to increased production of amyloids and chronic neuroinflammation, ultimately culminating in inflammaging.

PLWH with HAND have been shown to exhibit increased prevalence of CSF EVs, which carry cargoes for synapses, glial cells, inflammation, and stress responses compared with CSF EVs from uninfected controls [201]. A recent study from our lab has demonstrated increased numbers of neuronal-derived EVs (NDEVs) derived from the brain and serum of HIV-1 transgenic rats compared to wild-type controls [202]. Higher numbers of NDEVs have also been found in the plasma of PLWH with cognitive impairment and were found to be enriched in high-mobility group box 1 (HMGB1), neurofilament-light (NF-L), and Aβ. These studies thus suggest that NDEVs can serve as potential biomarkers for cognitive impairment in PLWH [203,204]. Additionally, these NDEVs were also shown to decrease in numbers with age in PLWH [204]. Several drugs of abuse have also been shown to potentiate HIV-1 infectivity (Figure 3). Therefore, understanding their role in HIV-1-induced aging will be an important area of research that warrants investigation. It has been reported that cocaine self-administration in mice reduced uptake of NDEVs by the astrocytes primarily in the motor cortex, an effect that was effectively reversed by extinction training [205]. Drugs of abuse like cocaine, morphine, methamphetamine [206,207,208] can induce chronic low-level inflammation and can also potentiate inflammatory status in the context of HIV-1 [209,210,211], which together can contribute to the process of inflammaging. As discussed above presence of ApoE4 is highly prevalent in the aging HIV-1 population. Interestingly, ApoE4 has also been shown to be associated with increased risk of Alzheimer’s disease [212]. Additionally, several studies have shown the deposition of amyloid β plaques and accumulation of neurotoxic amyloid proteins in the brains of PLWH on cART therapy [213,214,215], which can be a potential contributor to the process of inflammation-mediated aging. Although the role of glial cell-induced inflammation and inflammasomes in Alzheimer’s disease has been well studied [82,129,216,217,218,219], a recent study has demonstrated activation of neuronal NLRP1 by Aβ aggregates, and that, this activation cleaved caspase 1, which, in turn, leads to the production of matured IL1β and IL18. These cytokines together can induce the generation of caspase-6, leading to apoptosis and axonal degeneration [127]. It is likely that the packaging of NLRP1 in NDEVs could also lead to widespread neuroinflammation and is an area that warrants future investigation. Similar to Alzheimer’s disease, presence of amyloids also plays an important role in the process of neurodegeneration in PLWH on cART and can thus be considered as a co-morbidity of HIV-1 infection. Alzheimer’s disease-like pathology observed in PLWH can, in turn, lead to inflammaging either directly or via the EVs.

## 8. Conclusions and Future Perspectives

**Figure 4:**
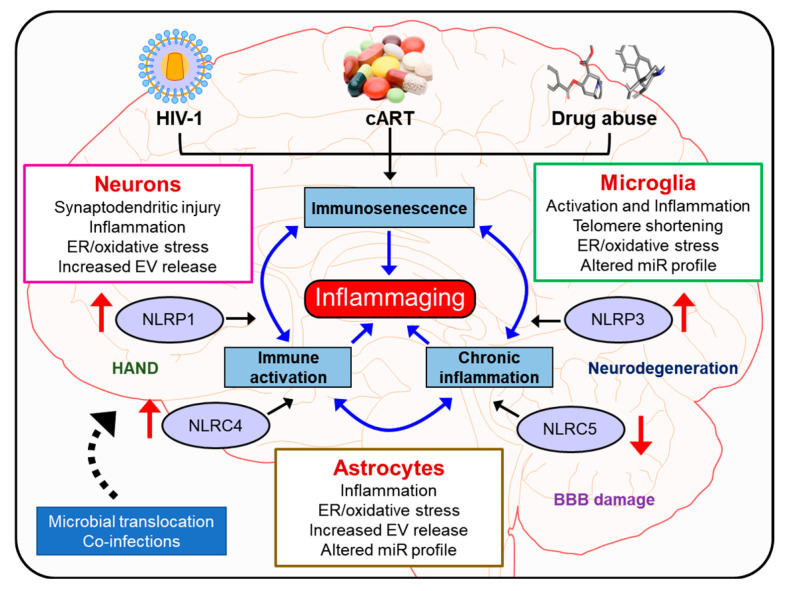
HIV-1/HIV-1 proteins, cART, and/or drug abuse induce inflammasome proteins via oxidative stress, altered miR expression, and EVs, thereby triggering glial activation, leading to secretion of proinflammatory cytokines resulting in synaptodendritic injury and neuroinflammation and ultimately neuroinflammaging.

Overall, HIV-1 and drug abuse play critical roles in inflammasome activation with ensuing neuroinflammation and neuroinflammaging in various CNS cells such as the microglia, astrocytes, and neurons (Figure 4). The development of brain-penetrating small molecules that can function as ROS scavengers, inflammasome modulators, and anti-inflammatory molecules that can dampen inflammasome signaling pathways could be considered as potential therapeutic strategies for restoring cognitive deficits associated with neurodegenerative disorders as well as neuroinflammaging. It should be cautioned that detailed mechanistic studies aimed at targeting specific cell types in the CNS are warranted before these molecules can enter the realm of clinical trials. In the future, it would be important to integrate the brain permeant signaling mediators of inflammasome signaling as adjunctive therapies with the current therapeutic archetypes for targeting neuroinflammaging.

## References

[1] Lohse N., Obel N. (2016). Update of Survival for Persons with HIV Infection in Denmark. Ann. Intern. Med..

[2] Legarth R.A., Ahlstrom M.G., Kronborg G., Larsen C.S., Pedersen C., Pedersen G., Mohey R., Gerstoft J., Obel N. (2016). Long-Term Mortality in HIV-Infected Individuals 50 Years or Older: A Nationwide, Population-Based Cohort Study. J. Acquir. Immune Defic. Syndr..

[3] Gross A.M., Jaeger P.A., Kreisberg J.F., Licon K., Jepsen K.L., Khosroheidari M., Morsey B.M., Swindells S., Shen H., Ng C.T. (2016). Methylome-wide Analysis of Chronic HIV Infection Reveals Five-Year Increase in Biological Age and Epigenetic Targeting of HLA. Mol. Cell..

[4] McArthur J.C., Johnson T.P. (2020). Chronic inflammation mediates brain injury in HIV infection: Relevance for cure strategies. Curr. Opin. Neurol..

[5] Deeks S.G. (2011). HIV infection, inflammation, immunosenescence, and aging. Annu. Rev. Med..

[6] Deeks S.G., Tracy R., Douek D.C. (2013). Systemic effects of inflammation on health during chronic HIV infection. Immunity.

[7] Leng S.X., Margolick J.B. (2019). Aging, sex, inflammation, frailty, and CMV and HIV infections. Cell. Immunol..

[8] High K.P., Brennan-Ing M., Clifford D.B., Cohen M.H., Currier J., Deeks S.G., Deren S., Effros R.B., Gebo K., Goronzy J.J. (2012). HIV and aging: State of knowledge and areas of critical need for research. A report to the NIH Office of AIDS Research by the HIV and Aging Working Group. J. Acquir. Immune Defic. Syndr..

[9] Guaraldi G., Prakash M., Moecklinghoff C., Stellbrink H.J. (2014). Morbidity in older HIV-infected patients: Impact of long-term antiretroviral use. Aids Rev..

[10] Steele A.K., Lee E.J., Vestal B., Hecht D., Dong Z., Rapaport E., Koeppe J., Campbell T.B., Wilson C.C. (2014). Contribution of intestinal barrier damage, microbial translocation and HIV-1 infection status to an inflammaging signature. PloS ONE.

[11] Sundermann E.E., Hussain M.A., Moore D.J., Horvath S., Lin D.T.S., Kobor M.S., Levine A., Group H. (2019). Inflammation-related genes are associated with epigenetic aging in HIV. J. Neurovirol..

[12] Palmer C.S., Palchaudhuri R., Albargy H., Abdel-Mohsen M., Crowe S.M. (2018). Exploiting immune cell metabolic machinery for functional HIV cure and the prevention of inflammaging. F1000Research.

[13] Kaplan-Lewis E., Aberg J.A., Lee M. (2017). Aging with HIV in the ART era. Semin. Diagn. Pathol..

[14] Nasi M., De Biasi S., Gibellini L., Bianchini E., Pecorini S., Bacca V., Guaraldi G., Mussini C., Pinti M., Cossarizza A. (2017). Ageing and inflammation in patients with HIV infection. Clin. Exp. Immunol..

[15] Xia C., Luo D., Yu X., Jiang S., Liu S. (2011). HIV-associated dementia in the era of highly active antiretroviral therapy (HAART). Microbes Infect..

[16] Singh D. (2012). What’s in a name? AIDS dementia complex, HIV-associated dementia, HIV-associated neurocognitive disorder or HIV encephalopathy. Afr. J. Psychiatry (Johannesbg.).

[17] Schouten J., Cinque P., Gisslen M., Reiss P., Portegies P. (2011). HIV-1 infection and cognitive impairment in the cART era: A review. AIDS.

[18] Aquaro S., Svicher V., Ronga L., Perno C.F., Pollicita M. (2008). HIV-1-associated dementia during HAART therapy. Recent Pat. Cns Drug Discov..

[19] Heaton R.K., Clifford D.B., Franklin D.R., Woods S.P., Ake C., Vaida F., Ellis R.J., Letendre S.L., Marcotte T.D., Atkinson J.H. (2010). HIV-associated neurocognitive disorders persist in the era of potent antiretroviral therapy: CHARTER Study. Neurology.

[20] Bonnet F., Amieva H., Marquant F., Bernard C., Bruyand M., Dauchy F.A., Mercie P., Greib C., Richert L., Neau D. (2013). Cognitive disorders in HIV-infected patients: Are they HIV-related?. AIDS.

[21] Elbirt D., Mahlab-Guri K., Bezalel-Rosenberg S., Gill H., Attali M., Asher I. (2015). HIV-associated neurocognitive disorders (HAND). Isr. Med. Assoc. J..

[22] Simioni S., Cavassini M., Annoni J.M., Rimbault Abraham A., Bourquin I., Schiffer V., Calmy A., Chave J.P., Giacobini E., Hirschel B. (2010). Cognitive dysfunction in HIV patients despite long-standing suppression of viremia. AIDS.

[23] Goodwin L.D., Goodwin W.L., Cantrill J.L. (1988). The mental health needs of elementary schoolchildren. J. Sch. Health.

[24] Cotto B., Natarajanseenivasan K., Langford D. (2019). HIV-1 infection alters energy metabolism in the brain: Contributions to HIV-associated neurocognitive disorders. Prog. Neurobiol..

[25] Bougea A., Spantideas N., Galanis P., Gkekas G., Thomaides T. (2019). Optimal treatment of HIV-associated neurocognitive disorders: Myths and reality. A critical review. Adv. Infect. Dis..

[26] Alfahad T.B., Nath A. (2013). Update on HIV-associated neurocognitive disorders. Curr. Neurol. Neurosci. Rep..

[27] Gelman B.B. (2015). Neuropathology of HAND With Suppressive Antiretroviral Therapy: Encephalitis and Neurodegeneration Reconsidered. Curr. Hiv/Aids Rep..

[28] Chan P., Hellmuth J., Spudich S., Valcour V. (2016). Cognitive Impairment and Persistent CNS Injury in Treated HIV. Curr. Hiv/Aids Rep..

[29] Marra C.M. (2015). HIV-associated neurocognitive disorders and central nervous system drug penetration: What next?. Antivir. Ther..

[30] Letendre S. (2011). Central nervous system complications in HIV disease: HIV-associated neurocognitive disorder. Top. Antivir. Med..

[31] Lanman T., Letendre S., Ma Q., Bang A., Ellis R. (2019). CNS Neurotoxicity of Antiretrovirals. J. Neuroimmunepharmacol.

[32] Dash S., Balasubramaniam M., Villalta F., Dash C., Pandhare J. (2015). Impact of cocaine abuse on HIV pathogenesis. Front. Microbiol.

[33] Tyagi M., Bukrinsky M., Simon G.L. (2016). Mechanisms of HIV Transcriptional Regulation by Drugs of Abuse. Curr. Hiv Res..

[34] Gurwell J.A., Nath A., Sun Q., Zhang J., Martin K.M., Chen Y., Hauser K.F. (2001). Synergistic neurotoxicity of opioids and human immunodeficiency virus-1 Tat protein in striatal neurons in vitro. Neuroscience.

[35] Turchan J., Anderson C., Hauser K.F., Sun Q., Zhang J., Liu Y., Wise P.M., Kruman I., Maragos W., Mattson M.P. (2001). Estrogen protects against the synergistic toxicity by HIV proteins, methamphetamine and cocaine. BMC Neurosci..

[36] Degenhardt L., Whiteford H., Hall W.D. (2014). The Global Burden of Disease projects: What have we learned about illicit drug use and dependence and their contribution to the global burden of disease?. Drug Alcohol. Rev..

[37] Degenhardt L., Whiteford H.A., Ferrari A.J., Baxter A.J., Charlson F.J., Hall W.D., Freedman G., Burstein R., Johns N., Engell R.E. (2013). Global burden of disease attributable to illicit drug use and dependence: Findings from the Global Burden of Disease Study 2010. Lancet.

[38] Holt J.L., Kraft-Terry S.D., Chang L. (2012). Neuroimaging studies of the aging HIV-1-infected brain. J. Neurovirol..

[39] Chang L., Shukla D.K. (2018). Imaging studies of the HIV-infected brain. Handb. Clin. Neurol..

[40] Vera J.H., Ridha B., Gilleece Y., Amlani A., Thorburn P., Dizdarevic S. (2017). PET brain imaging in HIV-associated neurocognitive disorders (HAND) in the era of combination antiretroviral therapy. Eur. J. Nucl. Med. Mol. Imaging.

[41] Campbell A.N., Tross S., Calsyn D.A. (2013). Substance use disorders and HIV/AIDS prevention and treatment intervention: Research and practice considerations. Soc. Work Public Health.

[42] Dahal S., Chitti S.V., Nair M.P., Saxena S.K. (2015). Interactive effects of cocaine on HIV infection: Implication in HIV-associated neurocognitive disorder and neuroAIDS. Front. Microbiol..

[43] Chang S.L., Connaghan K.P., Wei Y., Li M.D. (2014). NeuroHIV and use of addictive substances. Int. Rev. Neurobiol..

[44] Ene L. (2018). Human Immunodeficiency Virus in the Brain-Culprit or Facilitator?. Infect. Dis. (Auckl).

[45] Gougeon M.L. (2017). Alarmins and *Mol. Cell. Proteom* central nervous system inflammation in HIV-associated neurological disorders. J. Intern. Med..

[46] Hong S., Banks W.A. (2015). Role of the immune system in HIV-associated neuroinflammation and neurocognitive implications. Brain Behav. Immun..

[47] Clifford D.B., Ances B.M. (2013). HIV-associated neurocognitive disorder. Lancet Infect. Dis..

[48] Saylor D., Dickens A.M., Sacktor N., Haughey N., Slusher B., Pletnikov M., Mankowski J.L., Brown A., Volsky D.J., McArthur J.C. (2016). HIV-associated neurocognitive disorder—Pathogenesis and prospects for treatment. Nat. Rev. Neurol..

[49] Bell J.E. (2004). An update on the neuropathology of HIV in the HAART era. Histopathology.

[50] Gonzalez-Scarano F., Martin-Garcia J. (2005). The neuropathogenesis of AIDS. Nat. Rev. Immunol..

[51] Lawrence D.M., Major E.O. (2002). HIV-1 and the brain: Connections between HIV-1-associated dementia, neuropathology and neuroimmunology. Microbes Infect..

[52] Calder P.C., Bosco N., Bourdet-Sicard R., Capuron L., Delzenne N., Dore J., Franceschi C., Lehtinen M.J., Recker T., Salvioli S. (2017). Health relevance of the modification of low grade inflammation in ageing (inflammageing) and the role of nutrition. Ageing Res. Rev..

[53] Gruevska A., Moragrega A.B., Galindo M.J., Esplugues J.V., Blas-Garcia A., Apostolova N. (2020). p53 and p53-related mediators PAI-1 and IGFBP-3 are downregulated in peripheral blood mononuclear cells of HIV-patients exposed to non-nucleoside reverse transcriptase inhibitors. Antivir. Res..

[54] Lopez-Otin C., Blasco M.A., Partridge L., Serrano M., Kroemer G. (2013). The hallmarks of aging. Cell.

[55] Mackiewicz M.M., Overk C., Achim C.L., Masliah E. (2019). Pathogenesis of age-related HIV neurodegeneration. J. Neurovirol..

[56] Horvath S., Levine A.J. (2015). HIV-1 Infection Accelerates Age According to the Epigenetic Clock. J. Infect. Dis..

[57] Horvath S., Stein D.J., Phillips N., Heany S.J., Kobor M.S., Lin D.T.S., Myer L., Zar H.J., Levine A.J., Hoare J. (2018). Perinatally acquired HIV infection accelerates epigenetic aging in South African adolescents. AIDS.

[58] Johnson T.P., Patel K., Johnson K.R., Maric D., Calabresi P.A., Hasbun R., Nath A. (2013). Induction of IL-17 and nonclassical T-cell activation by HIV-Tat protein. Proc. Natl. Acad. Sci. USA.

[59] Lamkanfi M., Dixit V.M. (2012). Inflammasomes and their roles in health and disease. Annu. Rev. Cell Dev. Biol..

[60] Davis B.K., Wen H., Ting J.P. (2011). The inflammasome NLRs in immunity, inflammation, and associated diseases. Annu. Rev. Immunol..

[61] Kayagaki N., Warming S., Lamkanfi M., Vande Walle L., Louie S., Dong J., Newton K., Qu Y., Liu J., Heldens S. (2011). Non-canonical inflammasome activation targets caspase-11. Nature.

[62] Martinon F., Burns K., Tschopp J. (2002). The inflammasome: A molecular platform triggering activation of inflammatory caspases and processing of proIL-beta. Mol. Cell..

[63] Salvesen G.S., Dixit V.M. (1999). Caspase activation: The induced-proximity model. Proc. Natl. Acad. Sci. USA.

[64] Shi Y. (2004). Caspase activation: Revisiting the induced proximity model. Cell.

[65] Lamkanfi M., Kanneganti T.D., Van Damme P., Vanden Berghe T., Vanoverberghe I., Vandekerckhove J., Vandenabeele P., Gevaert K., Nunez G. (2008). Targeted peptidecentric proteomics reveals caspase-7 as a substrate of the caspase-1 inflammasomes. Mol. Cell. Proteom..

[66] Lamkanfi M., Declercq W., Vanden Berghe T., Vandenabeele P. (2006). Caspases leave the beaten track: Caspase-mediated activation of NF-kappaB. J. Cell Biol..

[67] Poyet J.L., Srinivasula S.M., Tnani M., Razmara M., Fernandes-Alnemri T., Alnemri E.S. (2001). Identification of Ipaf, a human caspase-1-activating protein related to Apaf-1. J. Biol. Chem..

[68] Faustin B., Lartigue L., Bruey J.M., Luciano F., Sergienko E., Bailly-Maitre B., Volkmann N., Hanein D., Rouiller I., Reed J.C. (2007). Reconstituted NALP1 inflammasome reveals two-step mechanism of caspase-1 activation. Mol. Cell.

[69] Duncan J.A., Bergstralh D.T., Wang Y., Willingham S.B., Ye Z., Zimmermann A.G., Ting J.P. (2007). Cryopyrin/NALP3 binds ATP/dATP, is an ATPase, and requires ATP binding to mediate inflammatory signaling. Proc. Natl. Acad. Sci. USA.

[70] Mariathasan S., Weiss D.S., Newton K., McBride J., O’Rourke K., Roose-Girma M., Lee W.P., Weinrauch Y., Monack D.M., Dixit V.M. (2006). Cryopyrin activates the inflammasome in response to toxins and ATP. Nature.

[71] Bouchier-Hayes L., Martin S.J. (2002). CARD games in apoptosis and immunity. Embo Rep..

[72] Strowig T., Henao-Mejia J., Elinav E., Flavell R. (2012). Inflammasomes in health and disease. Nature.

[73] Broz P., Dixit V.M. (2016). Inflammasomes: Mechanism of assembly, regulation and signalling. Nat Rev Immunol.

[74] Guo H., Callaway J.B., Ting J.P. (2015). Inflammasomes: Mechanism of action, role in disease, and therapeutics. Nat. Med..

[75] Lamkanfi M., Dixit V.M. (2014). Mechanisms and functions of inflammasomes. Cell.

[76] Walsh J.G., Muruve D.A., Power C. (2014). Inflammasomes in the CNS. Nat. Rev. Neurosci..

[77] Gustin A., Kirchmeyer M., Koncina E., Felten P., Losciuto S., Heurtaux T., Tardivel A., Heuschling P., Dostert C. (2015). NLRP3 Inflammasome Is Expressed and Functional in Mouse Brain Microglia but Not in Astrocytes. PloS ONE.

[78] Freeman L., Guo H., David C.N., Brickey W.J., Jha S., Ting J.P. (2017). NLR members NLRC4 and NLRP3 mediate sterile inflammasome activation in microglia and astrocytes. J. Exp. Med..

[79] Wu J., Fernandes-Alnemri T., Alnemri E.S. (2010). Involvement of the AIM2, NLRC4, and NLRP3 inflammasomes in caspase-1 activation by Listeria monocytogenes. J. Clin. Immunol..

[80] Minkiewicz J., de Rivero Vaccari J.P., Keane R.W. (2013). Human astrocytes express a novel NLRP2 inflammasome. Glia.

[81] Kaushal V., Dye R., Pakavathkumar P., Foveau B., Flores J., Hyman B., Ghetti B., Koller B.H., LeBlanc A.C. (2015). Neuronal NLRP1 inflammasome activation of Caspase-1 coordinately regulates inflammatory interleukin-1-beta production and axonal degeneration-associated Caspase-6 activation. Cell Death Differ..

[82] Saresella M., La Rosa F., Piancone F., Zoppis M., Marventano I., Calabrese E., Rainone V., Nemni R., Mancuso R., Clerici M. (2016). The NLRP3 and NLRP1 inflammasomes are activated in Alzheimer’s disease. Mol. Neurodegener..

[83] Kummer J.A., Broekhuizen R., Everett H., Agostini L., Kuijk L., Martinon F., van Bruggen R., Tschopp J. (2007). Inflammasome components NALP 1 and 3 show distinct but separate expression profiles in human tissues suggesting a site-specific role in the inflammatory response. J. Histochem. Cytochem..

[84] Hlaing T., Guo R.F., Dilley K.A., Loussia J.M., Morrish T.A., Shi M.M., Vincenz C., Ward P.A. (2001). Molecular cloning and characterization of DEFCAP-L and -S, two isoforms of a novel member of the mammalian Ced-4 family of apoptosis proteins. J. Biol. Chem..

[85] Levinsohn J.L., Newman Z.L., Hellmich K.A., Fattah R., Getz M.A., Liu S., Sastalla I., Leppla S.H., Moayeri M. (2012). Anthrax lethal factor cleavage of Nlrp1 is required for activation of the inflammasome. PloS Pathog..

[86] Chavarria-Smith J., Vance R.E. (2013). Direct proteolytic cleavage of NLRP1B is necessary and sufficient for inflammasome activation by anthrax lethal factor. PloS Pathog..

[87] Feria M.G., Taborda N.A., Hernandez J.C., Rugeles M.T. (2018). HIV replication is associated to inflammasomes activation, IL-1beta, IL-18 and caspase-1 expression in GALT and peripheral blood. PloS ONE.

[88] Lippai D., Bala S., Petrasek J., Csak T., Levin I., Kurt-Jones E.A., Szabo G. (2013). Alcohol-induced IL-1beta in the brain is mediated by NLRP3/ASC inflammasome activation that amplifies neuroinflammation. J. Leukoc. Biol..

[89] Lowe P.P., Cho Y., Tornai D., Coban S., Catalano D., Szabo G. (2020). Inhibition of the Inflammasome Signaling Cascade Reduces Alcohol Consumption in Female But Not Male Mice. Alcohol. Clin. Exp. Res..

[90] Zou J., Crews F.T. (2012). Inflammasome-IL-1beta Signaling Mediates Ethanol Inhibition of Hippocampal Neurogenesis. Front Neurosci..

[91] Hoffman H.M., Mueller J.L., Broide D.H., Wanderer A.A., Kolodner R.D. (2001). Mutation of a new gene encoding a putative pyrin-like protein causes familial cold autoinflammatory syndrome and Muckle-Wells syndrome. Nat. Genet..

[92] Vajjhala P.R., Mirams R.E., Hill J.M. (2012). Multiple binding sites on the pyrin domain of ASC protein allow self-association and interaction with NLRP3 protein. J. Biol. Chem..

[93] Walsh J.G., Reinke S.N., Mamik M.K., McKenzie B.A., Maingat F., Branton W.G., Broadhurst D.I., Power C. (2014). Rapid inflammasome activation in microglia contributes to brain disease in HIV/AIDS. Retrovirology.

[94] Mamik M.K., Hui E., Branton W.G., McKenzie B.A., Chisholm J., Cohen E.A., Power C. (2017). HIV-1 Viral Protein R Activates NLRP3 Inflammasome in Microglia: Implications for HIV-1 Associated Neuroinflammation. J. Neuroimmune Pharm..

[95] Guo H., Gao J., Taxman D.J., Ting J.P., Su L. (2014). HIV-1 infection induces interleukin-1beta production via TLR8 protein-dependent and NLRP3 inflammasome mechanisms in human monocytes. J. Biol. Chem..

[96] Chivero E.T., Guo M.L., Periyasamy P., Liao K., Callen S.E., Buch S. (2017). HIV-1 Tat Primes and Activates Microglial NLRP3 Inflammasome-Mediated Neuroinflammation. J. Neurosci..

[97] Pontillo A., Oshiro T.M., Girardelli M., Kamada A.J., Crovella S., Duarte A.J. (2012). Polymorphisms in inflammasome’ genes and susceptibility to HIV-1 infection. J. Acquir. Immune Defic. Syndr..

[98] Atluri V.S., Pilakka-Kanthikeel S., Garcia G., Jayant R.D., Sagar V., Samikkannu T., Yndart A., Nair M. (2016). Effect of Cocaine on HIV Infection and Inflammasome Gene Expression Profile in HIV Infected Macrophages. Sci. Rep..

[99] Franchi L., Amer A., Body-Malapel M., Kanneganti T.D., Ozoren N., Jagirdar R., Inohara N., Vandenabeele P., Bertin J., Coyle A. (2006). Cytosolic flagellin requires Ipaf for activation of caspase-1 and interleukin 1beta in salmonella-infected macrophages. Nat. Immunol..

[100] Miao E.A., Alpuche-Aranda C.M., Dors M., Clark A.E., Bader M.W., Miller S.I., Aderem A. (2006). Cytoplasmic flagellin activates caspase-1 and secretion of interleukin 1beta via Ipaf. Nat. Immunol..

[101] Ren T., Zamboni D.S., Roy C.R., Dietrich W.F., Vance R.E. (2006). Flagellin-deficient Legionella mutants evade caspase-1- and Naip5-mediated macrophage immunity. PloS Pathog..

[102] Dos Reis E.C., Leal V.N.C., Soares J., Fernandes F.P., Souza de Lima D., de Alencar B.C., Pontillo A. (2019). Flagellin/NLRC4 Pathway Rescues NLRP3-Inflammasome Defect in Dendritic Cells From HIV-Infected Patients: Perspective for New Adjuvant in Immunocompromised Individuals. Front Immunol..

[103] Kuenzel S., Till A., Winkler M., Hasler R., Lipinski S., Jung S., Grotzinger J., Fickenscher H., Schreiber S., Rosenstiel P. (2010). The nucleotide-binding oligomerization domain-like receptor NLRC5 is involved in IFN-dependent antiviral immune responses. J. Immunol..

[104] Lamkanfi M., Kanneganti T.D. (2012). Regulation of immune pathways by the NOD-like receptor NLRC5. Immunobiology.

[105] Benko S., Magalhaes J.G., Philpott D.J., Girardin S.E. (2010). NLRC5 limits the activation of inflammatory pathways. J. Immunol..

[106] Cui J., Zhu L., Xia X., Wang H.Y., Legras X., Hong J., Ji J., Shen P., Zheng S., Chen Z.J. (2010). NLRC5 negatively regulates the NF-kappaB and type I interferon signaling pathways. Cell.

[107] Hacker H., Karin M. (2006). Regulation and function of IKK and IKK-related kinases. Sci. Stke.

[108] Periyasamy P., Thangaraj A., Bendi V.S., Buch S. (2019). HIV-1 Tat-mediated microglial inflammation involves a novel miRNA-34a-NLRC5-NFkappaB signaling axis. Brain Behav. Immun..

[109] DiSabato D.J., Quan N., Godbout J.P. (2016). Neuroinflammation: The devil is in the details. J. Neurochem..

[110] Garvey L.J., Pavese N., Politis M., Ramlackhansingh A., Brooks D.J., Taylor-Robinson S.D., Winston A. (2014). Increased microglia activation in neurologically asymptomatic HIV-infected patients receiving effective ART. AIDS.

[111] Rubin L.H., Sacktor N., Creighton J., Du Y., Endres C.J., Pomper M.G., Coughlin J.M. (2018). Microglial activation is inversely associated with cognition in individuals living with HIV on effective antiretroviral therapy. AIDS.

[112] Vera J.H., Guo Q., Cole J.H., Boasso A., Greathead L., Kelleher P., Rabiner E.A., Kalk N., Bishop C., Gunn R.N. (2016). Neuroinflammation in treated HIV-positive individuals: A TSPO PET study. Neurology.

[113] Coughlin J.M., Wang Y., Ma S., Yue C., Kim P.K., Adams A.V., Roosa H.V., Gage K.L., Stathis M., Rais R. (2014). Regional brain distribution of translocator protein using [(11)C]DPA-713 PET in individuals infected with HIV. J. Neurovirol..

[114] Hammoud D.A., Endres C.J., Chander A.R., Guilarte T.R., Wong D.F., Sacktor N.C., McArthur J.C., Pomper M.G. (2005). Imaging glial cell activation with [11C]-R-PK11195 in patients with AIDS. J. Neurovirol..

[115] Alakkas A., Ellis R.J., Watson C.W., Umlauf A., Heaton R.K., Letendre S., Collier A., Marra C., Clifford D.B., Gelman B. (2019). White matter damage, neuroinflammation, and neuronal integrity in HAND. J. Neurovirol..

[116] Tavazzi E., Morrison D., Sullivan P., Morgello S., Fischer T. (2014). Brain inflammation is a common feature of HIV-infected patients without HIV encephalitis or productive brain infection. Curr. Hiv Res..

[117] Chao J., Zhang Y., Du L., Zhou R., Wu X., Shen K., Yao H. (2017). Molecular mechanisms underlying the involvement of the sigma-1 receptor in methamphetamine-mediated microglial polarization. Sci. Rep..

[118] Najera J.A., Bustamante E.A., Bortell N., Morsey B., Fox H.S., Ravasi T., Marcondes M.C. (2016). Methamphetamine abuse affects gene expression in brain-derived microglia of SIV-infected macaques to enhance inflammation and promote virus targets. BMC Immunol..

[119] Liao K., Guo M., Niu F., Yang L., Callen S.E., Buch S. (2016). Cocaine-mediated induction of microglial activation involves the ER stress-TLR2 axis. J. Neuroinflamm..

[120] Guo M.L., Liao K., Periyasamy P., Yang L., Cai Y., Callen S.E., Buch S. (2015). Cocaine-mediated microglial activation involves the ER stress-autophagy axis. Autophagy.

[121] Kettenmann H., Hanisch U.K., Noda M., Verkhratsky A. (2011). Physiology of microglia. Physiol. Rev..

[122] Harry G.J. (2013). Microglia during development and aging. Pharm. Ther..

[123] Chen N.C., Partridge A.T., Sell C., Torres C., Martin-Garcia J. (2017). Fate of microglia during HIV-1 infection: From activation to senescence?. Glia.

[124] Spudich S., Gonzalez-Scarano F. (2012). HIV-1-related central nervous system disease: Current issues in pathogenesis, diagnosis, and treatment. Cold Spring Harb. Perspect. Med..

[125] Lacagnina M.J., Rivera P.D., Bilbo S.D. (2017). Glial and Neuroimmune Mechanisms as Critical Modulators of Drug Use and Abuse. Neuropsychopharmacology.

[126] Gampierakis I.A., Koutmani Y., Semitekolou M., Morianos I., Charalampopoulos I., Xanthou G., Gravanis A., Karalis K.P. (2020). Hippocampal neural stem cells and microglia response to experimental inflammatory bowel disease (IBD). Mol. Psychiatry.

[127] Yap J.K.Y., Pickard B.S., Chan E.W.L., Gan S.Y. (2019). The Role of Neuronal NLRP1 Inflammasome in Alzheimer’s Disease: Bringing Neurons into the Neuroinflammation Game. Mol. Neurobiol..

[128] Poh L., Kang S.W., Baik S.H., Ng G.Y.Q., She D.T., Balaganapathy P., Dheen S.T., Magnus T., Gelderblom M., Sobey C.G. (2019). Evidence that NLRC4 inflammasome mediates apoptotic and pyroptotic microglial death following ischemic stroke. Brain Behav. Immun..

[129] Heneka M.T., McManus R.M., Latz E. (2018). Inflammasome signalling in brain function and neurodegenerative disease. Nat. Rev. Neurosci..

[130] Furman D., Chang J., Lartigue L., Bolen C.R., Haddad F., Gaudilliere B., Ganio E.A., Fragiadakis G.K., Spitzer M.H., Douchet I. (2017). Expression of specific inflammasome gene modules stratifies older individuals into two extreme clinical and immunological states. Nat. Med..

[131] Hu M.Y., Lin Y.Y., Zhang B.J., Lu D.L., Lu Z.Q., Cai W. (2019). Update of inflammasome activation in microglia/macrophage in aging and aging-related disease. Cns Neurosci..

[132] Scheiblich H., Trombly M., Ramirez A., Heneka M.T. (2020). Neuroimmune Connections in Aging and Neurodegenerative Diseases. Trends Immunol..

[133] Mecca C., Giambanco I., Donato R., Arcuri C. (2018). Microglia and Aging: The Role of the TREM2-DAP12 and CX3CL1-CX3CR1 Axes. Int. J. Mol. Sci..

[134] Cunningham C. (2013). Microglia and neurodegeneration: The role of systemic inflammation. Glia.

[135] Hickman S., Izzy S., Sen P., Morsett L., El Khoury J. (2018). Microglia in neurodegeneration. Nat. Neurosci..

[136] Lebeaupin C., Proics E., de Bieville C.H., Rousseau D., Bonnafous S., Patouraux S., Adam G., Lavallard V.J., Rovere C., Le Thuc O. (2015). ER stress induces NLRP3 inflammasome activation and hepatocyte death. Cell Death Dis..

[137] Zhou R., Yazdi A.S., Menu P., Tschopp J. (2011). A role for mitochondria in NLRP3 inflammasome activation. Nature.

[138] Kelley N., Jeltema D., Duan Y., He Y. (2019). The NLRP3 Inflammasome: An Overview of Mechanisms of Activation and Regulation. Int. J. Mol. Sci..

[139] Youm Y.H., Kanneganti T.D., Vandanmagsar B., Zhu X., Ravussin A., Adijiang A., Owen J.S., Thomas M.J., Francis J., Parks J.S. (2012). The Nlrp3 inflammasome promotes age-related thymic demise and immunosenescence. Cell Rep..

[140] Munoz-Planillo R., Kuffa P., Martinez-Colon G., Smith B.L., Rajendiran T.M., Nunez G. (2013). K(+) efflux is the common trigger of NLRP3 inflammasome activation by bacterial toxins and particulate matter. Immunity.

[141] Olivieri F., Rippo M.R., Monsurro V., Salvioli S., Capri M., Procopio A.D., Franceschi C. (2013). MicroRNAs linking inflamm-aging, cellular senescence and cancer. Ageing Res. Rev..

[142] Wang X.H., Wang T.L. (2018). MicroRNAs of microglia: Wrestling with central nervous system disease. Neural Regen. Res..

[143] Soreq H., Wolf Y. (2011). NeurimmiRs: microRNAs in the neuroimmune interface. Trends Mol. Med..

[144] Periyasamy P., Liao K., Kook Y.H., Niu F., Callen S.E., Guo M.L., Buch S. (2018). Cocaine-Mediated Downregulation of miR-124 Activates Microglia by Targeting KLF4 and TLR4 Signaling. Mol. Neurobiol..

[145] Liu J., Xu C., Chen L., Xu P., Xiong H. (2012). Involvement of Kv1.3 and p38 MAPK signaling in HIV-1 glycoprotein 120-induced microglia neurotoxicity. Cell Death Dis..

[146] Costa B.M., Yao H., Yang L., Buch S. (2013). Role of endoplasmic reticulum (ER) stress in cocaine-induced microglial cell death. J. Neuroimmune Pharm..

[147] Rawat P., Teodorof-Diedrich C., Spector S.A. (2019). Human immunodeficiency virus Type-1 single-stranded RNA activates the NLRP3 inflammasome and impairs autophagic clearance of damaged mitochondria in human microglia. Glia.

[148] Du L., Shen K., Bai Y., Chao J., Hu G., Zhang Y., Yao H. (2019). Involvement of NLRP3 inflammasome in methamphetamine-induced microglial activation through miR-143/PUMA axis. Toxicol. Lett..

[149] Lippai D., Bala S., Csak T., Kurt-Jones E.A., Szabo G. (2013). Chronic alcohol-induced microRNA-155 contributes to neuroinflammation in a TLR4-dependent manner in mice. PloS ONE.

[150] Wyczechowska D., Lin H.Y., LaPlante A., Jeansonne D., Lassak A., Parsons C.H., Molina P.E., Peruzzi F. (2017). A miRNA Signature for Cognitive Deficits and Alcohol Use Disorder in Persons Living with HIV/AIDS. Front. Mol. Neurosci..

[151] Chen Y., Swanson R.A. (2003). Astrocytes and brain injury. J. Cereb. Blood Flow Metab..

[152] Farina C., Aloisi F., Meinl E. (2007). Astrocytes are active players in cerebral innate immunity. Trends Immunol..

[153] Kielian T. (2006). Toll-like receptors in central nervous system glial inflammation and homeostasis. J. Neurosci. Res..

[154] Xiao C., Yang B.F., Asadi N., Beguinot F., Hao C. (2002). Tumor necrosis factor-related apoptosis-inducing ligand-induced death-inducing signaling complex and its modulation by c-FLIP and PED/PEA-15 in glioma cells. J. Biol. Chem..

[155] Song J.H., Bellail A., Tse M.C., Yong V.W., Hao C. (2006). Human astrocytes are resistant to Fas ligand and tumor necrosis factor-related apoptosis-inducing ligand-induced apoptosis. J. Neurosci..

[156] Cohen J., Torres C. (2019). Astrocyte senescence: Evidence and significance. Aging Cell.

[157] Matias I., Morgado J., Gomes F.C.A. (2019). Astrocyte Heterogeneity: Impact to Brain Aging and Disease. Front. Aging Neurosci..

[158] Zhang J., Jiang N., Zhang L., Meng C., Zhao J., Wu J. (2020). NLRP6 expressed in astrocytes aggravates neurons injury after OGD/R through activating the inflammasome and inducing pyroptosis. Int. Immunopharmacol..

[159] Voet S., Srinivasan S., Lamkanfi M., van Loo G. (2019). Inflammasomes in neuroinflammatory and neurodegenerative diseases. Embo Mol. Med..

[160] Park J.H., Choi J.Y., Jo C., Koh Y.H. (2020). Involvement of ADAM10 in acrolein-induced astrocytic inflammation. Toxicol. Lett..

[161] Hong Y., Liu Y., Yu D., Wang M., Hou Y. (2019). The neuroprotection of progesterone against Abeta-induced NLRP3-Caspase-1 inflammasome activation via enhancing autophagy in astrocytes. Int. Immunopharmacol..

[162] Pacheco A.L., Vicentini G., Matteucci K.C., Ribeiro R.R., Weinlich R., Bortoluci K.R. (2019). The impairment in the NLRP3-induced NO secretion renders astrocytes highly permissive to T. cruzi replication. J. Leukoc. Biol..

[163] Sun Y.B., Zhao H., Mu D.L., Zhang W., Cui J., Wu L., Alam A., Wang D.X., Ma D. (2019). Dexmedetomidine inhibits astrocyte pyroptosis and subsequently protects the brain in in vitro and in vivo models of sepsis. Cell Death Dis..

[164] Ojeda D.S., Grasso D., Urquiza J., Till A., Vaccaro M.I., Quarleri J. (2018). Cell Death Is Counteracted by Mitophagy in HIV-Productively Infected Astrocytes but Is Promoted by Inflammasome Activation among Non-productively Infected Cells. Front. Immunol..

[165] Ebrahimi T., Rust M., Kaiser S.N., Slowik A., Beyer C., Koczulla A.R., Schulz J.B., Habib P., Bach J.P. (2018). Alpha1-Antitrypsin mitigates NLRP3-inflammasome activation in amyloid beta1-42-stimulated murine astrocytes. J. Neuroinflamm..

[166] Cheon S.Y., Kim E.J., Kim S.Y., Kim J.M., Kam E.H., Park J.K., Koo B.N. (2018). Apoptosis Signal-regulating Kinase 1 Silencing on Astroglial Inflammasomes in an Experimental Model of Ischemic Stroke. Neuroscience.

[167] Slowik A., Lammerding L., Zendedel A., Habib P., Beyer C. (2018). Impact of steroid hormones E2 and P on the NLRP3/ASC/Casp1 axis in primary mouse astroglia and BV-2 cells after in vitro hypoxia. J. Steroid. Biochem. Mol. Biol..

[168] de Paula Martins R., Ghisoni K., Lim C.K., Aguiar A.S., Guillemin G.J., Latini A. (2018). Neopterin preconditioning prevents inflammasome activation in mammalian astrocytes. Free Radic. Biol. Med..

[169] Du R.H., Wu F.F., Lu M., Shu X.D., Ding J.H., Wu G., Hu G. (2016). Uncoupling protein 2 modulation of the NLRP3 inflammasome in astrocytes and its implications in depression. Redox Biol..

[170] Liu S., Li Q., Zhang M.T., Mao-Ying Q.L., Hu L.Y., Wu G.C., Mi W.L., Wang Y.Q. (2016). Curcumin ameliorates neuropathic pain by down-regulating spinal IL-1beta via suppressing astroglial NALP1 inflammasome and JAK2-STAT3 signalling. Sci. Rep..

[171] Jian Z., Ding S., Deng H., Wang J., Yi W., Wang L., Zhu S., Gu L., Xiong X. (2016). Probenecid protects against oxygen-glucose deprivation injury in primary astrocytes by regulating inflammasome activity. Brain Res..

[172] Alfonso-Loeches S., Urena-Peralta J.R., Morillo-Bargues M.J., Oliver-De La Cruz J., Guerri C. (2014). Role of mitochondria ROS generation in ethanol-induced NLRP3 inflammasome activation and cell death in astroglial cells. Front. Cell Neurosci..

[173] Liu L., Chan C. (2014). IPAF inflammasome is involved in interleukin-1beta production from astrocytes, induced by palmitate; implications for Alzheimer’s Disease. Neurobiol. Aging.

[174] Silverman W.R., de Rivero Vaccari J.P., Locovei S., Qiu F., Carlsson S.K., Scemes E., Keane R.W., Dahl G. (2009). The pannexin 1 channel activates the inflammasome in neurons and astrocytes. J. Biol. Chem..

[175] Bhat R., Crowe E.P., Bitto A., Moh M., Katsetos C.D., Garcia F.U., Johnson F.B., Trojanowski J.Q., Sell C., Torres C. (2012). Astrocyte senescence as a component of Alzheimer’s disease. PloS ONE.

[176] Chinta S.J., Lieu C.A., Demaria M., Laberge R.M., Campisi J., Andersen J.K. (2013). Environmental stress, ageing and glial cell senescence: A novel mechanistic link to Parkinson’s disease?. J. Intern. Med..

[177] Kang C., Xu Q., Martin T.D., Li M.Z., Demaria M., Aron L., Lu T., Yankner B.A., Campisi J., Elledge S.J. (2015). The DNA damage response induces inflammation and senescence by inhibiting autophagy of GATA4. Science.

[178] Yu C., Narasipura S.D., Richards M.H., Hu X.T., Yamamoto B., Al-Harthi L. (2017). HIV and drug abuse mediate astrocyte senescence in a beta-catenin-dependent manner leading to neuronal toxicity. Aging Cell.

[179] Churchill M.J., Wesselingh S.L., Cowley D., Pardo C.A., McArthur J.C., Brew B.J., Gorry P.R. (2009). Extensive astrocyte infection is prominent in human immunodeficiency virus-associated dementia. Ann. Neurol..

[180] Barat C., Proust A., Deshiere A., Leboeuf M., Drouin J., Tremblay M.J. (2018). Astrocytes sustain long-term productive HIV-1 infection without establishment of reactivable viral latency. Glia.

[181] Li G.H., Henderson L., Nath A. (2016). Astrocytes as an HIV Reservoir: Mechanism of HIV Infection. Curr. Hiv Res..

[182] Li G.H., Maric D., Major E.O., Nath A. (2020). Productive HIV infection in astrocytes can be established via a nonclassical mechanism. AIDS.

[183] Al-Harti L., Joseph J., Nath A. (2018). Astrocytes as an HIV CNS reservoir: Highlights and reflections of an NIMH-sponsored symposium. J. Neurovirol..

[184] Zhao X., Fan Y., Vann P.H., Wong J.M., Sumien N., He J.J. (2020). Long-term HIV-1 Tat Expression in the Brain Led to Neurobehavioral, Pathological, and Epigenetic Changes Reminiscent of Accelerated Aging. Aging Dis..

[185] Cohen J., D’Agostino L., Wilson J., Tuzer F., Torres C. (2017). Astrocyte Senescence and Metabolic Changes in Response to HIV Antiretroviral Therapy Drugs. Front. Aging Neurosci..

[186] Gonzalez H., Podany A., Al-Harthi L., Wallace J. (2020). The far-reaching HAND of cART: cART effects on astrocytes. J. Neuroimmune Pharm..

[187] Dang J., Tiwari S.K., Agrawal K., Hui H., Qin Y., Rana T.M. (2020). Glial cell diversity and methamphetamine-induced neuroinflammation in human cerebral organoids. Mol. Psychiatry.

[188] Shi Y., Yuan S., Tang S.J. (2019). Morphine and HIV-1 gp120 cooperatively promote pathogenesis in the spinal pain neural circuit. Mol. Pain.

[189] Chang L., Andres M., Sadino J., Jiang C.S., Nakama H., Miller E., Ernst T. (2011). Impact of apolipoprotein E epsilon4 and HIV on cognition and brain atrophy: Antagonistic pleiotropy and premature brain aging. Neuroimage.

[190] Chang L., Jiang C., Cunningham E., Buchthal S., Douet V., Andres M., Ernst T. (2014). Effects of APOE epsilon4, age, and HIV on glial metabolites and cognitive deficits. Neurology.

[191] Evans S., Dowell N.G., Tabet N., Tofts P.S., King S.L., Rusted J.M. (2014). Cognitive and neural signatures of the APOE E4 allele in mid-aged adults. Neurobiol. Aging.

[192] Crawford F., Wood M., Ferguson S., Mathura V., Gupta P., Humphrey J., Mouzon B., Laporte V., Margenthaler E., O’Steen B. (2009). Apolipoprotein E-genotype dependent hippocampal and cortical responses to traumatic brain injury. Neuroscience.

[193] Theendakara V., Peters-Libeu C.A., Spilman P., Poksay K.S., Bredesen D.E., Rao R.V. (2016). Direct Transcriptional Effects of Apolipoprotein E. J. Neurosci..

[194] Babu H., Ambikan A.T., Gabriel E.E., Svensson Akusjarvi S., Palaniappan A.N., Sundaraj V., Mupanni N.R., Sperk M., Cheedarla N., Sridhar R. (2019). Systemic Inflammation and the Increased Risk of Inflamm-Aging and Age-Associated Diseases in People Living With HIV on Long Term Suppressive Antiretroviral Therapy. Front. Immunol..

[195] Zhang Y., Kim M.S., Jia B., Yan J., Zuniga-Hertz J.P., Han C., Cai D. (2017). Hypothalamic stem cells control ageing speed partly through exosomal miRNAs. Nature.

[196] Olivieri F., Albertini M.C., Orciani M., Ceka A., Cricca M., Procopio A.D., Bonafe M. (2015). DNA damage response (DDR) and senescence: Shuttled inflamma-miRNAs on the stage of inflamm-aging. Oncotarget.

[197] Urbanelli L., Buratta S., Sagini K., Tancini B., Emiliani C. (2016). Extracellular Vesicles as New Players in Cellular Senescence. Int. J. Mol. Sci..

[198] Xu D., Tahara H. (2013). The role of exosomes and microRNAs in senescence and aging. Adv. Drug Deliv. Rev..

[199] Bertoldi K., Cechinel L.R., Schallenberger B., Corssac G.B., Davies S., Guerreiro I.C.K., Bello-Klein A., Araujo A.S.R., Siqueira I.R. (2018). Circulating extracellular vesicles in the aging process: Impact of aerobic exercise. Mol. Cell Biochem..

[200] Eitan E., Green J., Bodogai M., Mode N.A., Baek R., Jorgensen M.M., Freeman D.W., Witwer K.W., Zonderman A.B., Biragyn A. (2017). Age-Related Changes in Plasma Extracellular Vesicle Characteristics and Internalization by Leukocytes. Sci. Rep..

[201] Guha D., Lorenz D.R., Misra V., Chettimada S., Morgello S., Gabuzda D. (2019). Proteomic analysis of cerebrospinal fluid extracellular vesicles reveals synaptic injury, inflammation, and stress response markers in HIV patients with cognitive impairment. J. Neuroinflamm..

[202] Dagur R.S., Liao K., Sil S., Niu F., Sun Z., Lyubchenko Y.L., Peeples E.S., Hu G., Buch S. (2020). Neuronal-derived extracellular vesicles are enriched in the brain and serum of HIV-1 transgenic rats. J. Extracell. Vesicles.

[203] Pulliam L., Sun B., Mustapic M., Chawla S., Kapogiannis D. (2019). Plasma neuronal exosomes serve as biomarkers of cognitive impairment in HIV infection and Alzheimer’s disease. J. Neurovirol..

[204] Sun B., Dalvi P., Abadjian L., Tang N., Pulliam L. (2017). Blood neuron-derived exosomes as biomarkers of cognitive impairment in HIV. AIDS.

[205] Jarvis R., Tamashiro-Orrego A., Promes V., Tu L., Shi J., Yang Y. (2019). Cocaine Self-administration and Extinction Inversely Alter Neuron to Glia Exosomal Dynamics in the Nucleus Accumbens. Front. Cell Neurosci..

[206] de Guglielmo G., Fu Y., Chen J., Larrosa E., Hoang I., Kawamura T., Lorrai I., Zorman B., Bryant J., George O. (2020). Increases in compulsivity, inflammation, and neural injury in HIV transgenic rats with escalated methamphetamine self-administration under extended-access conditions. Brain Res..

[207] Sil S., Periyasamy P., Guo M.L., Callen S., Buch S. (2018). Morphine-Mediated Brain Region-Specific Astrocytosis Involves the ER Stress-Autophagy Axis. Mol. Neurobiol..

[208] Sil S., Niu F., Tom E., Liao K., Periyasamy P., Buch S. (2019). Cocaine Mediated Neuroinflammation: Role of Dysregulated Autophagy in Pericytes. Mol. Neurobiol..

[209] Volpe G.E., Ward H., Mwamburi M., Dinh D., Bhalchandra S., Wanke C., Kane A.V. (2014). Associations of cocaine use and HIV infection with the intestinal microbiota, microbial translocation, and inflammation. J. Stud. Alcohol Drugs.

[210] Reiner B.C., Keblesh J.P., Xiong H. (2009). Methamphetamine abuse, HIV infection, and neurotoxicity. Int. J. Physiol. Pathophysiol. Pharm..

[211] Dave R.S. (2012). Morphine affects HIV-induced inflammatory response without influencing viral replication in human monocyte-derived macrophages. Fems Immunol. Med. Microbiol..

[212] Strittmatter W.J., Weisgraber K.H., Huang D.Y., Dong L.M., Salvesen G.S., Pericak-Vance M., Schmechel D., Saunders A.M., Goldgaber D., Roses A.D. (1993). Binding of human apolipoprotein E to synthetic amyloid beta peptide: Isoform-specific effects and implications for late-onset Alzheimer disease. Proc. Natl. Acad. Sci. USA.

[213] Xu J., Ikezu T. (2009). The comorbidity of HIV-associated neurocognitive disorders and Alzheimer’s disease: A foreseeable medical challenge in post-HAART era. J. Neuroimmune Pharm..

[214] Achim C.L., Adame A., Dumaop W., Everall I.P., Masliah E., Neurobehavioral Research C. (2009). Increased accumulation of intraneuronal amyloid beta in HIV-infected patients. J. Neuroimmune Pharm..

[215] Sil S., Hu G., Liao K., Niu F., Callen S., Periyasamy P., Fox H.S., Buch S. (2020). HIV-1 Tat-mediated astrocytic amyloidosis involves the HIF-1alpha/lncRNA BACE1-AS axis. Plos Biol..

[216] Liu L., Chan C. (2014). The role of inflammasome in Alzheimer’s disease. Ageing Res. Rev..

[217] Sweeney M.D., Ayyadurai S., Zlokovic B.V. (2016). Pericytes of the neurovascular unit: Key functions and signaling pathways. Nat. Neurosci..

[218] Bell R.D., Winkler E.A., Sagare A.P., Singh I., LaRue B., Deane R., Zlokovic B.V. (2010). Pericytes control key neurovascular functions and neuronal phenotype in the adult brain and during brain aging. Neuron.

[219] Nelson A.R., Sagare M.A., Wang Y., Kisler K., Zhao Z., Zlokovic B.V. (2020). Channelrhodopsin Excitation Contracts Brain Pericytes and Reduces Blood Flow in the Aging Mouse Brain in vivo. Front. Aging Neurosci..

